# First-Pass Success of Tracheal Intubation With Videolaryngoscopy in Head and Neck Cancer Patients: A Registry-Based Retrospective Cohort Study

**DOI:** 10.7759/cureus.20857

**Published:** 2021-12-31

**Authors:** Faisal Shamim, Ausaf A Khan, Fauzia A Khan

**Affiliations:** 1 Anaesthesiology, Aga Khan University, Karachi, PAK

**Keywords:** videolaryngoscopy, intubation, head and neck cancer, database, airway management

## Abstract

Background

The incidence of difficult airway is higher in head and neck oncological surgery than in other surgeries. Limited evidence is available on the use of videolaryngoscopes in this cohort. A registry database on perioperative management of these patients was set up in our department in 2017.

Methods

Data from 2018 to 2019 were retrieved from this database. In 128 patients, videolaryngoscopy was used as the initial airway management of choice. Ease of intubation by first-pass success, its association with accessory manoeuvres, and complications were noted.

Results

Of the patients, 87% (n = 111) were successfully intubated with a videolaryngoscope in the first attempts. There was a strong association between the use of external laryngeal manipulation and successful first-pass intubation with videolaryngoscope. In patients with reduced inter-incisor distance, videolaryngoscope has shown greater benefit. There were very few complications including bleeding from the tumour site and a transient decrease in oxygen saturation to 88% in two patients.

Conclusion

Videolaryngoscopy was associated with high first-attempt intubation success and we recommend its use as the initial choice for airway management in head and neck cancer patients.

## Introduction

Oral cancer is ranked as the sixth most common cancer worldwide [1] and 40% occur in Southeast Asian countries (India, Sri Lanka, Bangladesh, and Pakistan) [2]. The incidence of difficult intubation is higher in patients with ear, nose, and throat malignancies than in the general surgical population (15.75% vs. 2.5%) [3,4].

The success of conventional laryngoscopy routinely depends on adequate mouth opening, proper head positioning, and consistent anatomy [5]. In head and neck cancer patients, limited mouth opening, poor tissue mobility, airway distortion, and prior radiotherapy increase the failure rate of tracheal intubation with conventional laryngoscopy [6]. Videolaryngoscopy (VL) has improved the success of tracheal intubation in difficult airway management as it produces a view of the laryngeal inlet independent of the line of sight. Limited evidence exists on the use of videolaryngoscope in head and neck cancer [6]. Previous studies that have shown successful intubation with VL in the predicted difficult airway have not included patients with head and neck pathology, including malignancies or a history of head and neck surgery or radiation.

Aga Khan University Hospital is a tertiary care referral centre for head and neck cancer surgery in Pakistan. Videolaryngoscope is commonly used in our practice for predicted difficult airways. Department of Anaesthesiology developed a registry database for perioperative anaesthetic management in head and neck cancer surgery in 2017. The purpose of creating this particular database was to utilize pooled data with particular emphasis on airway management in head and neck cancer surgery and integrate all relevant information for improvement in patient outcomes.

This study aimed to evaluate the use of videolaryngoscope in patients who presented for head and neck cancer surgery with regards to success, failure, and intubation-related complications. The primary objective was to assess the ease of intubation by first-pass success with VL. Additionally, we observed the number of attempts, use of accessory manoeuvres, and adjuncts to facilitate intubation.

This article was previously presented as a meeting abstract at the International Guy’s Airway Management Webcast 2021 (online course version) on 29th January 2021.

## Materials and methods

**Figure 1:**
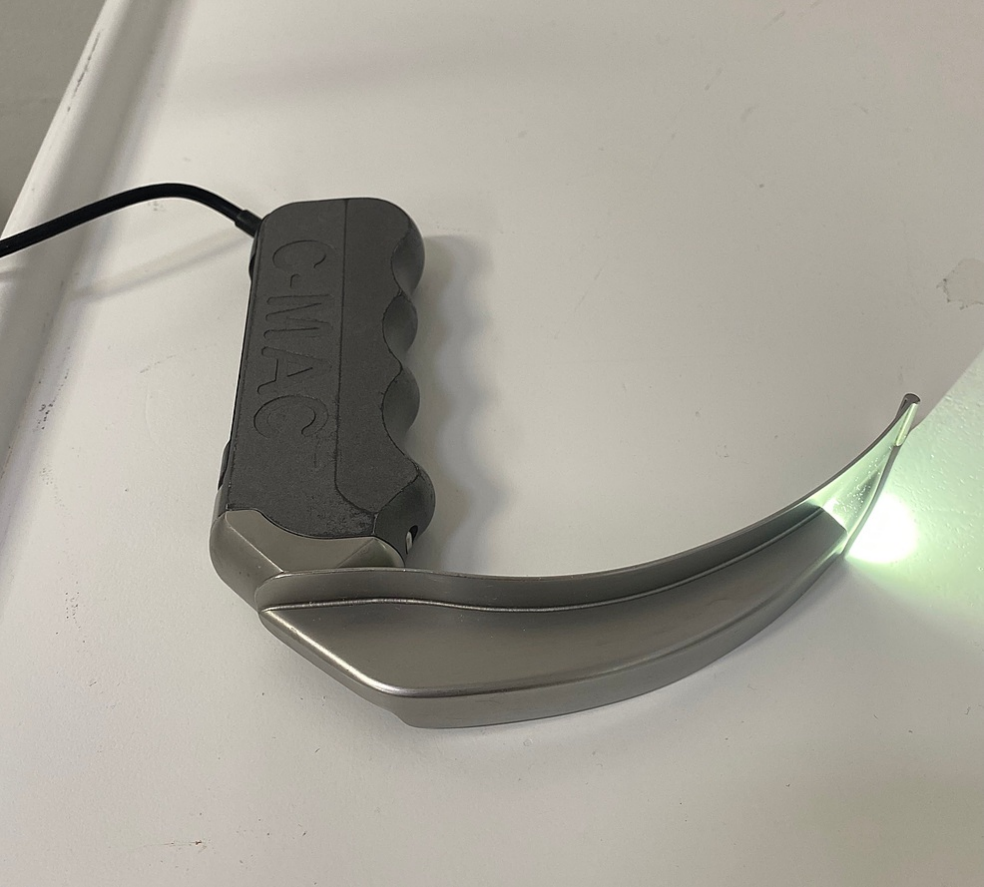
C-MAC videolaryngoscope with Macintosh type blade size 4.

This was a retrospective cohort study with data drawn from a perioperative registry database in anaesthetic management for head and neck cancer surgical patients. Approval of this study was provided by the Ethical Review Committee of Aga Khan University (2020-3500-8487) on 16th February 2020. We searched our database from 1st January 2018 to 31st December 2019. We included all adult patients, 18 years and above, who underwent head and neck cancer surgery in whom videolaryngoscope was used as a primary device for airway management. During the study period, we were only using the C-MAC videolaryngoscope (Karl Storz, Tuttlingen, Germany) with Macintosh type blade size 4 (Figure 1).

Sedation premedication was avoided in all patients due to predicted difficult airway. Patients in whom tracheostomy or fiberoptic intubation was performed as the primary technique of airway management were excluded. Standard American Society of Anesthesiologists (ASA) monitoring included pulse oximetry, electrocardiography, non-invasive blood pressure, and end-tidal carbon dioxide measurements.

A predefined template for data extraction was used. The following variables were retrieved from the database: age, gender, weight, height, ASA physical status classification, and comorbidities. Airway assessment parameters included Mallampati classification, inter-incisor distance (IID), thyromental distance (TD), upper lip bite test (ULBT), and neck movements. The IID and TD were objectively measured by a ruler. Route of intubation and type of tracheal tube used was noted. Ease of intubation was defined as adequate glottis visualization and intubation accomplished in the first attempt [7]. Intubation success was defined as confirmation of endotracheal tube (ETT) placement by end-tidal carbon dioxide monitoring with a single blade insertion [8]. Use of accessory manoeuvres such as external laryngeal manipulation or Magill forceps and intubation aids like tracheal tube stylet or bougie was noted. Complications, particularly hypoxemia (oxygen saturation [SpO2] < 90%), bleeding from the tumour site, and failed intubation, were extracted from the database. This manuscript was prepared according to the Strengthening the Reporting of Observational Studies in Epidemiology (STROBE) statement.

Qualitative and quantitative point estimates were computed as the frequency with percentage and mean (standard deviation [SD]) or median (interquartile range [IQR]), respectively. Continuous data were compared using Student's t‐test and categorical data using the chi‐square test. For univariate analysis, unadjusted odds ratios were calculated, and for multivariate analysis, backward stepwise logistic regression was performed. A p-value < 0.05 was considered statistically significant. Analyses were performed using Statistical Package for the Social Sciences (SPSS) version 19 (IBM Corp., Armonk, NY).

## Results

A total of 325 patients underwent head and neck cancer surgery under general anaesthesia during the study period. In 128 patients, the videolaryngoscope was used as the primary device for tracheal intubation. Table 1 shows patients demographics, the origin of head and neck pathology, anaesthetic technique, and intubation details.

**Table 1:** Patient demographics, origin of head and neck pathology, anaesthetic technique, and intubation details (n = 128). ASA, American Society of Anesthesiologists; COPD, chronic obstructive pulmonary disease.

Characteristics		Point estimates
Age (years, mean [SD])		50.70 ± 12.97
Weight (kg, mean [SD])		70.52 ± 16.93
Height (cm, mean [SD])		164.24 ± 9.24
Gender	Male	99 (77.3%)
	Female	29 (22.7%)
ASA	I	8 (6.3%)
	II	99 (77.3%)
	II	21 (16.4%)
Comorbidities	Hypertension	39 (30.5%)
	Diabetes mellitus	26 (20.3%)
	COPD/asthma	7 (5.5%)
	Anaemia	7 (5.5%)
	Addiction/smoker	20 (15.6%)
	Others	12 (9.4%)
Induction technique	Intravenous	116 (90.62%)
	Inhalational	12 (9.37%)
Use of muscle relaxant	Cisatracurium	93 (72.65%)
	Succinylcholine	23 (17.96%)
	No muscle relaxant	12 (9.37%)
Route of intubation	Nasal	106 (82.8%)
	Oral	20 (15.6%)
Origin of head and neck cancer/pathology	Oral cavity	115 (89.84%)
	Larynx	6 (4.68%)
	External	5 (3.90%)
	Pharynx	2 (1.56)

The average IID was 2.53 cm (SD: 1.11; range: 0.2-6) and TD was 6.86 cm (SD 1.73; range: 3-14). The results of IID, Mallampati classification, ULBT, TD, alteration of airway management technique, use of external laryngeal manipulation, and use of Magill forceps are given in Table 2.

**Table 2:** Ease of intubation with respect to airway assessment characteristics (n = 128).

Variables		Total	Intubation	
			First attempt (n = 111)	≥2 attempts (n = 17)
Inter-incisor distance	≤1.5	28	22 (78.6%)	6 (21.4%)
	>1.5	100	89 (89.0%)	11 (11.0%)
Mallampati classification	I & II	28	85 (85%)	15 (15%)
	III & IV	100	26 (92.9%)	2 (7.1%)
Upper lip bite test	III	27	25 (92.6%)	2 (7.4%)
	I & II	101	86 (85.1%)	15 (14.9%)
Thyromental distance	≤6 cm	47	41 (87.2%)	6 (12.8%)
	>6cm	81	70 (86.4%)	11 (13.6%)
Alteration of airway management	Yes	19	14 (73.7%)	5 (26.3%)
	No	109	97 (89%)	12 (11%)
External laryngeal manipulation	Yes	64	54 (84.4%)	10 (15.6%)
	No	64	57 (89.1%)	7 (10.9%)
Use of Magill forceps	Yes	22	15 (68.2%)	7 (31.8%)
	No	106	96 (90.6%)	10 (9.4%)

Of the patients, 87% (n = 111) were successfully intubated with a videolaryngoscope in the first attempts, whereas 11.7% (n = 15) were intubated in the second attempt. Out of 128 patients, two (1.56%) needed alteration of airway management technique and the airway was secured by tracheostomy. Of the patients, 50% required external laryngeal manipulation in addition to the use of a videolaryngoscope.

In univariate analysis, the unadjusted odds ratio of IID (<1.5 cm), Mallampati classification (III and IV), and need for external laryngeal manipulation showed a negative but insignificant relationship with ease of intubation. The UBLT (grade III) and TD (≤6 cm) showed a positive relationship with ease of intubation but the result did not reach statistical significance. Only the use of Magill forceps was negatively and significantly associated with ease of intubation (OR = 0.22; 95% CI: 0.07-0.67) (Table 3). In multivariable analysis, backward stepwise logistic regression was performed. Adjusted odds ratio showing the effect of alteration airway (p = 0.037) and use of Magill forceps (p = 0.004) were negatively associated with ease of intubation after controlling the other explanatory variables in the model. Interaction of external pressure required and IID (≤1.5 cm) were also negatively associated with ease of intubation by videolaryngoscope (Table 3).

**Table 3:** Univariate and multivariable analyses for factors associated with ease of intubation with videolaryngoscopy. * significant at p < 0.05; ** significant at p < 0.01.

Factors	Unadjusted odds ratio (95% CI)	P-value	Adjusted odds ratio (95% CI)	P-value
Inter-incisor distance (cm)	0.45 (0.15-1.36)	0.158	-	-
Mallampati classification	0.43 (0.09-2.03)	0.29	0.29 (0.05-1.69)	0.172
Upper lip bite test	2.18 (0.46-10.18)	0.32	5.41 (0.91-31.99)	0.063
Alteration airway	0.35 (0.11-1.13)	0.08	0.18 (0.04-0.90)	0.037*
External pressure required	0.66 (0.23-1.87)	0.44	4.19 (0.82-21.53)	0.086
Use of Magill forceps	0.22 (0.07-0.67)	0.008**	0.11 (0.01-0.57)	0.004**
Thyromental distance	1.07 (0.37-3.12)	0.89	-	-
Neck movement	1.41 (0.16-11.91)	0.75	-	-
Interaction external pressure required x inter-incisor distance	-		0.09 (0.01-0.57)	0.011*

Complications were observed in nine (7.2%) cases during or immediately after tracheal intubation. One patient had bleeding from tumour site in the oral cavity, six patients exhibited significant haemodynamic response (more than 20% of baseline) to laryngoscopy, which was of relatively longer duration compared to others, and there was decreased oxygen saturation up to 88% in two patients.

## Discussion

In this study, videolaryngoscope using Macintosh type blade in head and neck cancer patients was associated with a first-pass intubation success rate of 89% in patients with an IID of > 1.5 cm and 79% in patients with IID less than 1.5 cm. All patients were successfully intubated within two attempts except two who required tracheostomy due to failed intubation. As per details found in the registry database, one patient had significant bleeding from mass at the base of the tongue, causing glottic view obscure, which resulted in three failed attempts. The other patient had limited mouth opening (IID: 1.5 cm) and a high anterior larynx resulting in a steep path for ETT passage through vocal cords. Bougie was also tried but was not successful, and after multiple attempts, both the anaesthetist and surgeon decided to do a tracheostomy. The normal IID is greater than 3.5 cm. Our results have highlighted that even in reduced IID of less than 1.5 cm, the use of external laryngeal manipulation with VL can facilitate nasotracheal intubation without significant major complications.

A total of 27 patients with IID less than 1.5 cm also had Mallampati class III and IV and ULBT class III. In the presence of predicted difficult airway, videolaryngoscope has been beneficial. In many patients with head and neck cancer, mouth opening is limited, and it is not possible to perform Mallampati or ULBT. In such cases, IID can be used as a predictor of success in tracheal intubation. Further studies and data may be required to validate this observation. Traditionally, flexible bronchoscopy has been used in patients with limited mouth opening [8]. Though videolaryngoscope has become popular, it is a common belief that they are not of much use in these patients. In low middle-income countries (LMIC) particularly, budgetary constraints may only favour buying one instrument to deal with a difficult airway. In our experience, VL is more robust compared to flexible bronchoscope, which is more costly and requires relatively more service time and repair cost if damaged.

Aziz et al. compared C-MAC and Macintosh laryngoscope in suspected difficult airway scenarios [9] in 300 patients with various predicted difficult airways. Skilled providers achieved higher success rates for tracheal intubation on the first attempt with C-MAC video laryngoscope (93%) than direct laryngoscopy (84%). The use of a gum-elastic bougie and/or external laryngeal manipulation was required less often in the C-MAC intubations (24%, 33/138) compared with direct laryngoscopy (37%, 46/124; p = 0.020). Their study revealed a higher success rate with C-MAC, and lesser use of adjuncts and external manoeuvres in the C-MAC group; however, the failure rate was not different between the two despite a good glottic view. Their study population did not identify patients with difficult airways due to head and neck cancer. In our literature search, we have found only one randomized controlled trial that included patients with head and neck cancer. Hazarika et al. compared the C-MAC D-blade videolaryngoscope and the standard Macintosh laryngoscope for nasal intubation in patients with head and neck cancer [10]. The time required for intubation was less in the VL group (39.56 ± 15.65 s), the success rate was 100% (50/50) with C-MAC D-blade versus 84% (42/50), and ease of intubation was more in the VL group than in the Macintosh group (p < 0.05).

The strength of our study is that it has provided data from real-life situations in patients with a difficult airway. Several authors have used mannikins or simulated difficult airways to find the best technique. Kleine-Brueggeney et al. compared six videolaryngoscopes in 720 patients with simulated difficult airway by application of a cervical collar to permit a minimal mouth opening of 18 mm, and the head was taped to the trolley to inhibit neck movement with the primary outcome of first-attempt intubation success rate [11]. They concluded that McGrath (Aircraft Medical Ltd, Edinburgh, UK) and C-MAC D-blade showed the highest success rates and lowest rates of tissue trauma but also identified their limitation that this trial studied simulated difficult airways and conclusions regarding genuine difficult airways must be drawn with caution.

There are some limitations of our study. Firstly, it is a single-centre study, so our results may not be generalizable. Secondly, the database did not record the level of the anaesthesiologist intubating the trachea, so the experience and competence of performing physicians may influence the results; however, in our institutional practice, an experienced consultant anaesthesiologist is always present inside the operating room for anaesthetic induction of difficult airway patient. Thirdly, the study, being retrospective, was not designed to examine outcomes (reduced morbidity and mortality) or cost-effectiveness.

## Conclusions

Although the current evidence still has gaps for recommending this technique to be considered as a standard of care, videolaryngoscopes are being increasingly used and considered first-line intubation devices for difficult, rescue, and routine intubations, achieving the same or higher success rate.

In conclusion, we found that videolaryngoscopy using Macintosh type blade is a useful device for intubation in head and neck cancer patients with decreased inter-incisor distance less than normal.

## References

[1] Shah JP, Gil Z (2009). Current concepts in management of oral cancer - surgery. Oral Oncol.

[2] Anwer AW, Faisal M, Malik AA, Jamshed A, Hussain R, Pirzada MT (2018). Head and neck cancer in a developing country- a hospital based retrospective study across 10 years from Pakistan. J Cancer Allied Spec.

[3] Ahmed-Nusrath A (2017). Anaesthesia for head and neck cancer surgery. BJA Educ.

[4] Wong P, Iqbal R, Light Light, KP KP, Williams E, Hayward J (2016). Head and neck surgery in a tertiary centre: predictors of difficult airway and anaesthetic management. Proc Singapore Healthc.

[5] Myatra SN (2019). Optimal position for laryngoscopy - time for individualization?. J Anaesthesiol Clin Pharmacol.

[6] Assefa S, Sahile WA (2021). Anesthesia for head and neck surgery. Ethiop J Health Sci.

[7] Tosh P, Rajan S, Kumar L (2018). Ease of Intubation with C-MAC videolaryngoscope: use of 60° angled styletted endotracheal tube versus intubation over bougie. Anesth Essays Res.

[8] Collins SR, Blank RS (2014). Fiberoptic intubation: an overview and update. Respir Care.

[9] Aziz MF, Dillman D, Fu R, Brambrink AM (2012). Comparative effectiveness of the C-MAC video laryngoscope versus direct laryngoscopy in the setting of the predicted difficult airway. Anesthesiology.

[10] Hazarika H, Saxena A, Meshram P, Kumar Bhargava A (2018). A randomized controlled trial comparing C Mac D Blade and Macintosh laryngoscope for nasotracheal intubation in patients undergoing surgeries for head and neck cancer. Saudi J Anaesth.

[11] Kleine-Brueggeney M, Greif R, Schoettker P, Savoldelli GL, Nabecker S, Theiler LG (2016). Evaluation of six videolaryngoscopes in 720 patients with a simulated difficult airway: a multicentre randomized controlled trial. Br J Anaesth.

